# Exploring weaning-related shock: Cardiac injury and overload, platelet-endothelial cell activation, and inflammatory response markers following ECMO decannulation

**DOI:** 10.1177/02676591261439583

**Published:** 2026-04-06

**Authors:** Lara C.A. Pladet, Carlos V. Elzo Kraemer, Stefan Nierkens, Jeannine A. Hermens, Emma Rademaker, Lennie P.G. Derde, Christiaan L. Meuwese, Dirk W. Donker, Olaf L. Cremer

**Affiliations:** 1 Department of Intensive Care Medicine, University Medical Center Utrecht, Utrecht, The Netherlands; 2 Department of Intensive Care Medicine, Leiden University Medical Center, Leiden, The Netherlands; 3 Center for Translational Immunology, University Medical Center Utrecht, Utrecht, The Netherlands; 4 Princess Máxima Center for Pediatric Oncology, Utrecht, The Netherlands; 5 Julius Center, University Medical Center Utrecht, Utrecht, The Netherlands; 6 Department of Intensive Care, Erasmus Medical Center Rotterdam, Rotterdam, The Netherlands; 7 Cardiovascular and Respiratory Physiology, TechMed Center, University of Twente, Enschede, The Netherlands

**Keywords:** extracorporeal membrane oxygenation, cardiac overload, inflammation, platelet-endothelial cell activation, weaning

## Abstract

**Introduction:**

Mortality in patients receiving Extracorporeal Membrane Oxygenation (ECMO) is partly attributable to unanticipated hemodynamic instability after elective decannulation, termed weaning-related shock (WRS). This study explored the etiology of WRS by analyzing sequential plasma biomarker profiles.

**Methods:**

We analyzed a prospective cohort (2011–2022) of consecutive patients weaned from veno-arterial (V-A) or veno-venous (V-V) ECMO to evaluate WRS occurrence. WRS was defined as a rise in Vasoactive Inotropic Score ≥15 points for >6 h with a positive fluid balance and lactate >2 mmol/L within 72 h post-decannulation. Shock etiology was adjudicated as hemorrhagic, cardiogenic, septic, or unclassifiable. Subsequently, key plasma biomarkers reflecting inflammation, platelet-endothelial cell activation, and cardiac injury and overload were assessed pre- and post-decannulation, stratified on WRS occurrence, apparent etiology, and ECMO mode.

**Results:**

Out of 97 eligible individuals, 88 (91%) were analyzed (70 V-A and 18 V-V). Among them, 24 (27%) patients developed WRS, all of whom had received V-A ECMO. WRS was attributed to hemorrhagic, cardiogenic, septic, or unclassifiable causes in 0, 8, 8 and 8 cases, respectively. No differences were observed between groups in markers of inflammation or platelet-endothelial cell activation, nor in their trajectories over time. Conversely, NT-proBNP levels, but not HsTnI, increased significantly after decannulation in WRS patients compared to V-V or V-A ECMO patients without WRS (*p* = 0.003 and *p* = 0.02, respectively). NT-proBNP was also higher in these subjects prior to decannulation (all *p* < 0.001).

**Conclusion:**

Biomarker responses following ECMO decannulation suggest cardiac overload as the primary driver of WRS, irrespective of clinical shock diagnosis.

## Introduction

Veno-venous (V-V) and veno-arterial (V-A) extracorporeal membrane oxygenation (ECMO) are increasingly being used as a last-resort mechanical support for patients with severe refractory respiratory and/or cardiocirculatory failure.^1^ Despite this, mortality rates remain high up to around 50%, depending on ECMO mode and indication.^2^ Although most deaths occur while on ECMO, a substantial number of patients decease after elective weaning of support.^2,3^ The etiology of these late deaths, which are commonly referred to as the “ECMO gap”,^4,5^ is multifactorial. Yet, it seems in part related to the occurrence of weaning-related shock (WRS), a phenomenon characterized by unanticipated and severe hemodynamic instability shortly after decannulation.^6,7,8^

Clinically, new-onset fever and vasoplegia are commonly observed in this context, leading to the hypothesis that new or worsening inflammation or infection may contribute to WRS.^6,7,9^ Alternatively, weaning from V-A ECMO could unmask or provoke progression of underlying heart failure.^10^ However, despite its great clinical significance, the etiology of WRS remains largely elusive.^4,5^

Improved understanding of the pathophysiological processes underlying WRS is essential to develop novel therapeutic targets as well as prognostic biomarkers. Furthermore, distinguishing between septic, cardiogenic, and hemorrhagic shock is essential to initiate an appropriate treatment response. In this exploratory study we investigated the potential etiologies of WRS by examining sequential plasma biomarkers reflecting inflammation, platelet–endothelial cell adhesion, and cardiac injury and overload responses during the period immediately before and after ECMO decannulation.

## Materials and methods

### Design

This study was conducted at the intensive care unit (ICU) of the University Medical Center Utrecht, a tertiary academic medical center. We analyzed patients who were enrolled both in the Prediction of Weanability, Survival and Functional Outcomes after ECLS (PRECISE-ECLS) cohort (NCT05444764) and the Molecular Diagnosis and Risk Stratification of Sepsis (MARS) cohort (NCT01905033). The latter comprises a biorepository of daily plasma samples prospectively collected from ICU patients since 2011 using a standardized protocol. The institutional medical ethics committee granted approval for an opt-out method of consent for both studies (reference numbers 21-604/C and 10-056).

### Patient population

Consecutive patients who had been weaned from ECMO between 2011 and 2022 because of (presumed) convalescence were analyzed. We included both V-A and V-V supported patients, anticipating that WRS could occur in both groups, as sepsis and/or systemic have been implicated as important and frequent contributors to its pathogenesis.^6^ Patients were excluded if ECMO was terminated for reasons other than organ recovery, such as palliative care initiation, heart or lung transplantation, or long-term mechanical assist device implantation. Immunocompromised patients were also excluded from analysis to exclude confounding effects of immunosuppression on the biomarkers analyzed.

Structured algorithms for ECMO weaning were implemented in our center in 2018 (details available in Supplemental file 1). In short, V-A ECMO weaning commenced after >24 h of hemodynamic stability (pulse pressure >10 mmHg, MAP >65 mmHg, lactate < 1.5 mmol/L, SVO_2_ > 65%) with minimal vasopressor support, favorable recovery etiology, euvolemia, resolved pulmonary edema, and adequate liver function. Weaning trials then proceeded with echocardiographic monitoring during flow reduction to ≤1.5 L/min, ensuring LVEF >20–25%, LVOT-VTI >10 cm, and lateral mitral annulus velocity >6 cm/sec. For V-V ECMO, weaning required plateau pressures <30 cmH_2_O and FiO_2_ <0.4, followed by 24 h of zero sweep gas with maintained gas exchange before decannulation.

### Data collection

The MARS biorepository includes prospectively adjudicated data on patient and disease characteristics, with particular emphasis on infectious events. These data were supplemented with ECMO-specific information from PRECISE-ECLS, a bidirectional cohort initiated in 2021, which combines both prospectively and retrospectively collected datafrom electronic health records. The occurrence of WRS was defined as1^1^ an increase of ≥15 points in the Vasoactive Inotropic Score (VIS) for at least 6 hours, together with both^2^ a positive 24-h cumulative fluid balance of >1 L and^3^ at least a single measured lactate >2 mmol/L, all occurring within the first 72 h after liberation of ECMO. Hypotension with the necessity of high dosages vasopressant and/or inotropic medication reflects impaired systemic perfusion, while elevated lactate levels indicate tissue hypoperfusion and anaerobic metabolism. A positive fluid balance in WRS was required to avoid the inadvertent inclusion of subjects receiving vasopressors as part of a deresuscitation strategy. In patients who met this case definition, we additionally adjudicated the tentative (main) cause of shock based on the clinical information available at the time. These included (1) hemorrhagic shock, defined as a bleeding complication necessitating the transfusion of ≥3 units of packed red blood cells within a 24 h period or requiring surgical or radiological intervention (i.e., in accordance with ELSO registry criteria^11^); (2) cardiogenic shock, as defined by the need for vasopressive or inotropic medication and/or mechanical support (i.e., in accordance with Society for Cardiovascular Angiography and Interventions (SCAI) criteria stage C, D and E,^12^ (3) septic shock, defined as the start of empirical antibiotic treatment for a clinically suspected new-onset infection in a setting of increasing hemodynamic compromise using the MARS database (which independently and prospectively adjudicated infectious events in all ICU patients from 2011 onwards),^13^ and (4) unclassifiable in case the clinical reason for WRS could not clearly be attributed to one of the above causes.

In addition, all measured C-Reactive Protein (CRP) levels, leukocyte counts and temperatures within the 72-h period before and after decannulation were collected to assess new-onset clinical inflammation following ECMO cessation. This was defined as ≥1 of the following signs: a temperature drop to <36 or rise to >38°C (with >1°C change), leukocytes drop to <4 or increase to >12 × 10^9/L (with >1 change), and CRP rise to >100 (with a >20 change).

### Biomarker measurements

Key biomarkers of inflammation, platelet-endothelial cell activation, and cardiac injury and overload were selected based on literature.^14,15^ These included:

(1) **Inflammatory markers:** high-sensitive C-Reactive Protein (hs-CRP) mg/L, Interferon-gamma (INF-y) pg/mL, INF-α pg/mL, INF-β pg/mL, Interferon gamma-induced Protein 10 (IP-10) pg/mL, Tumor Necrosis Factor-Related Apoptosis-Inducing Ligand (TRAIL) pg/mL, Monocyte Chemoattractant Protein-1 (MCP1) pg/mL, Macrophage Inflammatory Protein-1 alpha (MIP-1 α) pg/mL.
(2) **Platelet-endothelial cell activation markers**: Endothelial Selectin (E-Selectin) ng/mL, Platelet Selectin (P-Selectin) ng/mL, Intercellular Adhesion Molecule-3 (ICAM-3) ng/mL.
(3) **Cardiac injury and overload markers**: High-sensitive Cardiac Troponin I (HsTnI) pg/mL and N-terminal pro-brain Natriuretic Peptide (NT-proBNP) pg/mL.

For established biomarkers that have been validated for clinical use (i.e., hs-CRP, HsTnI and NT-proBNP) we used standard diagnostic assays in heparinized plasma samples, whereas we used the Meso Scale Discovery (MSD) platform in potassium-ethylenediaminetetraacetic acid samples for all other biomarkers. These were evaluated at four timepoints: approximately one day and immediately prior to ECMO decannulation (T - 1 and T0), as well as one and two days post-decannulation (T + 1 and T + 2) (Supplement Figure 1(a)). We performed before/after measurements twice to reduce variability in the MSD assay results. When concentrations were below the lower limit of quantitation, their values were imputed at half that threshold level. Biomarkers validated for clinical use were analyzed only at T0 and T + 1 (Supplement Figure 1(b)). As samples in the MARS biorepository had been collected as part of a routine daily workflow, any missingness was likely due to logistical reasons and not related to WRS occurrence. Moreover, since most biomarkers were measured in duplicate, the risk of bias from missing samples was deemed low.

### Data analysis

We used descriptive statistics to summarize the data according to ECMO mode, WRS occurrence, and presumed WRS etiology, reporting medians and interquartile range (IQR). Differences between groups were assessed using Chi-square tests. Biomarker trajectories were visually inspected using line plots and boxplots, if appropriate. Changes in biomarker levels from T - 1/T0 to T + 1/T + 2 were compared between patients with and without WRS using Mann-Whitney U tests. We applied separate Benjamini-Hochberg corrections for multiple testing to control the false discovery rate within each panel of inflammatory, platelet-endothelial cell activation, and cardiac injury and overload markers.^16^ Lastly, a sensitivity analysis was performed to assess whether the association between WRS occurrence and observed NT-proBNP concentrations was affected by the concurrent presence of AKI. Statistical significance was defined as a *p*-value <0.05. All analyses were performed using R version 4.2.2.

## Results

Among 97 patients eligible for study inclusion, 88 (91%) subjects were analyzed, including 70 V-A and 18 V-V ECMO cases (see flowchart Figure 1). A total of 24 (27%) patients developed WRS, all within the V-A ECMO group. WRS occurrence in V-A ECMO patients was associated with higher ICU mortality (*n* = 13, 54%) compared with both V-A ECMO patients without WRS (*n* = 9, 20%) and V-V ECMO patients (*n* = 1, 6%) (*p* = 0.009 and *p* = 0.004, respectively). Among those who ultimately died in the ICU, median time from ECMO weaning to death was 7 (IQR 4–12) days for WRS patients, compared to 15 (IQR 6–27) days for non-WRS patients.

**Figure 1.**
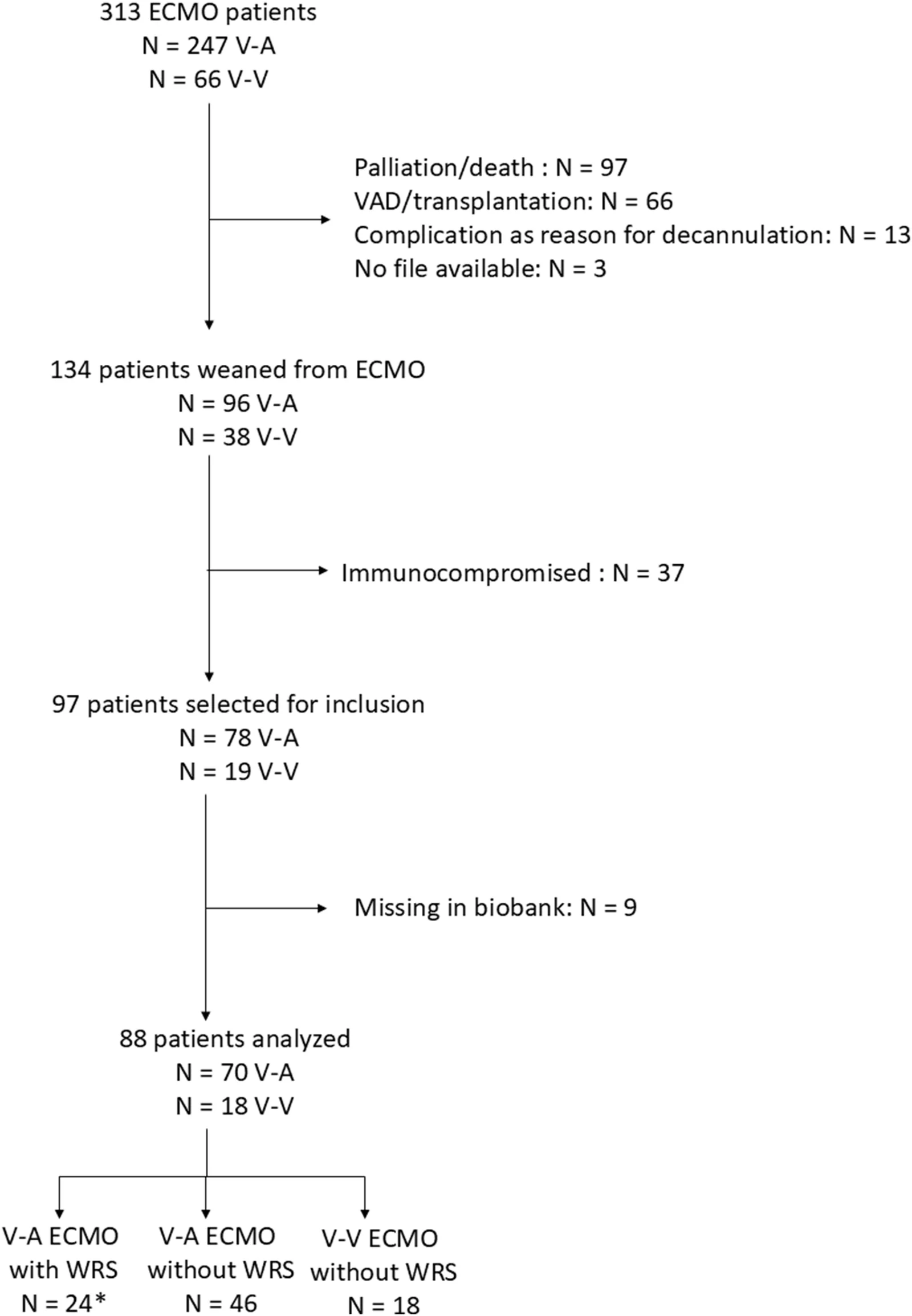
Flowchart of included patients *of 24 patients with weaning related shock, a total of three patients were recannulated, of which two survived and one deceased.

Following clinical adjudication, WRS was believed to be attributable to hemorrhagic, cardiogenic, and septic shock in 0 (0%), 8 (33%) and 8 (33%) cases, respectively. The remaining 8 (33%) cases remained unclassifiable. V-A ECMO duration and cumulative fluid balance prior to weaning did not differ significantly between patients with and without WRS (*p* = 0.21 and *p* = 0.053, respectively). WRS occurred more frequently in older patients (*p* < 0.01), those with diabetes mellitus (*p* = 0.02) and in subjects who had received postcardiotomy ECMO support (*p* < 0.01 (Table 1). Among these postcardiotomy patients, WRS occurred in 3 of 8 patients (38%) who underwent isolated coronary artery bypass grafting surgery, and 13 of 25 patients (52%) who underwent valve, aortic root or combined procedures (supplemental Table 1). Patients with WRS more frequently received inotropic support at the time of decannulation than those without WRS (dobutamine use 36% versus 5%, and milrinone use 45% versus 19%, respectively.

**Table 1.** Patient and treatment characteristics stratified by ECMO mode and the occurrence of weaning-related shock.

	No WRS occurrence		WRS occurrence
Demographics	V-V ECMO (*N* = 18)	V-A ECMO (*N* = 46)	V-A ECMO (*N* = 24)
Age (median ([IQR])	49 [32–58]	59 [49–70]	69 [60–74]
Male sex (*N*, %)	10 (56)	33 (72)	18 (75)
Diabetes mellitus (*N*, %)	0 (0)	8 (17)	8 (33)
Hypertension (*N*, %)	3 (17)	15 (33)	12 (50)
Heart failure (*N*, %)	1 (6)	8 (17)	6 (25)
Cerebrovascular accident (*N*, %)	0 (0)	2 (4)	2 (8)
Chronic kidney disease (*N*, %)	1 (6)	2 (4)	3 (13)
Chronic obstructive pulmonary disease (*N*, %)	1 (6)	2 (4)	0 (0)
ICU admission			
Apache IV score (median [IQR])	69 [59–83]	69 [50–86]	75 [62–94]
Pre ECMO cardiac arrest (*N*, %)	0 (0)	19 (41)	11 (46)
ECMO indication (*N*, %)			
Severe respiratory failure	12 (67)	0 (0)	0 (0)
Acute myocardial infarction	0 (0)	10 (22)	5 (21)
Cardiomyopathy	0 (0)	6 (13)	1 (4)
ECPR	0 (0)	10 (22)	2 (8)
Post-cardiotomy support	0 (0)	17 (37)	16 (67)
Other	6 (33)	3 (7)	0 (0)
Fluid balance (ml) prior to weaning	−69.50 [−440.50–233.75]	604.50 [-979.00–1815.75]	1828 [293.25–3067.50]
Duration of ECMO support in days ((median [IQR])	12 [7–15]	4 [2–7]	6 [3–10]

ECPR = ExtraCorporeal Cardiopulmonary Resuscitation.

In the first 72 h after decannulation, we observed an overall increase in CRP and temperature, but not leukocytes (Figure 2). New-onset clinical inflammation (rise in CRP, leukocytes, and/or new fever) was observed in 66 (75%) patients overall, including 22 (92%) V-A patients with WRS versus 36 (78%) V-A patients without WRS (*p* = 0.84), and 8 (44%) V-V patients without WRS (*p* = 0.01). Among WRS patients, new-onset clinical inflammation occurred at similar rates across all subgroups of cardiogenic (*n* = 6, 75%), septic (*n* = 7, 88%) and unclassifiable shock (*n* = 8, 100%) (*p* = 0.32).

**Figure 2.**
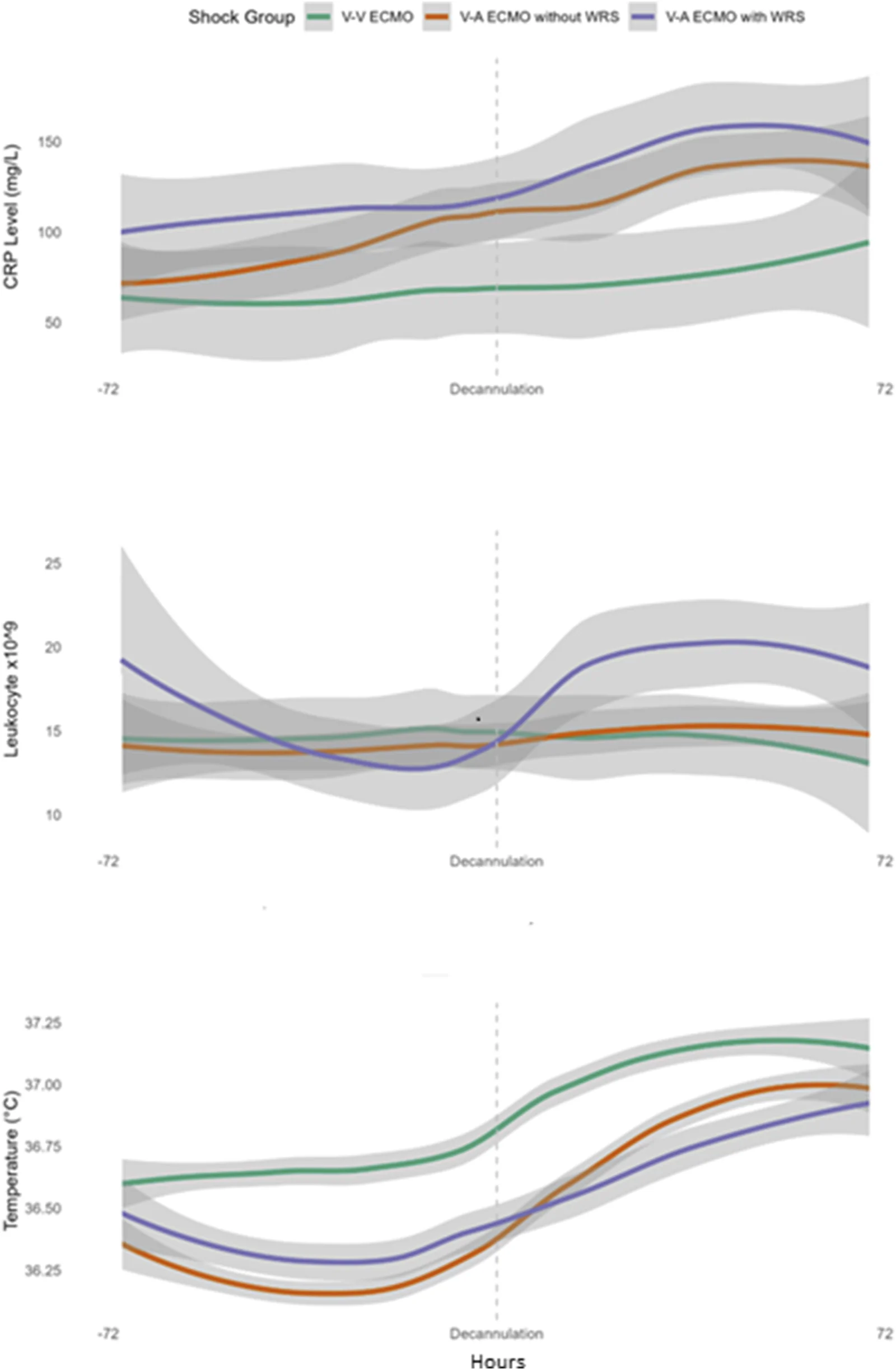
C-Reactive Protein (CRP), leukocyte counts and temperatures surrounding decannulation stratified by WRS occurrence Lines represent locally estimated scatterplot smoothing to depict trends in measured data. Grey shaded area represents the 95% confidence interval. 124 × 190 mm (96 × 96 DPI).

We observed elevated plasma concentrations for hs-CRP, IP-10, MCP-1, and P-selectin, and suppressed TRAIL levels compared to the reference ranges for these biomarkers in healthy individuals, at all timepoints, across all groups (Figure 3(a)–(b)). However, we did not observe any differences in trends for these biomarkers over time when comparing patients with and without WRS (*p* > 0.05 for all between-group comparisons)**.** Likewise, elevated levels of HsTnI were observed in V-A (but not V-V) ECMO patients compared to reference standards in healthy individuals. However, no differences in HsTnI trajectories between patients with and without WRS were observed after decannulation (*p* > 0.05 for all between-group comparisons) (Figure 3(c)). In contrast, NT-proBNP levels rose significantly more after decannulation in WRS patients compared to V-V and V-A ECMO patients who did not develop WRS (*p* = 0.003 and *p* = 0.02, respectively). However, NT-proBNP values were already higher in WRS patients prior to decannulation (*p* < 0.001 for all between-group comparisons) (Figure 3). These observations were robust in a sensitivity analysis that excluded patients who already had established AKI at time of decannulation (Supplement Figure 2). Strikingly, the rise in NT-proBNP levels was most pronounced not in patients with clinically suspected cardiogenic shock, but in those with presumed septic or unclassifiable shock (Supplement Figure 3). After introduction of weaning protocols in 2018, there was a significant decrease in cumulative fluid balance prior to weaning (median of +845 ml before 2018 and a median of +85 ml thereafter, *p* = 0.04), but no significant differences in the incidence of WRS (Supplement Table 2, *p* = 0.1).

**Figure 3.**
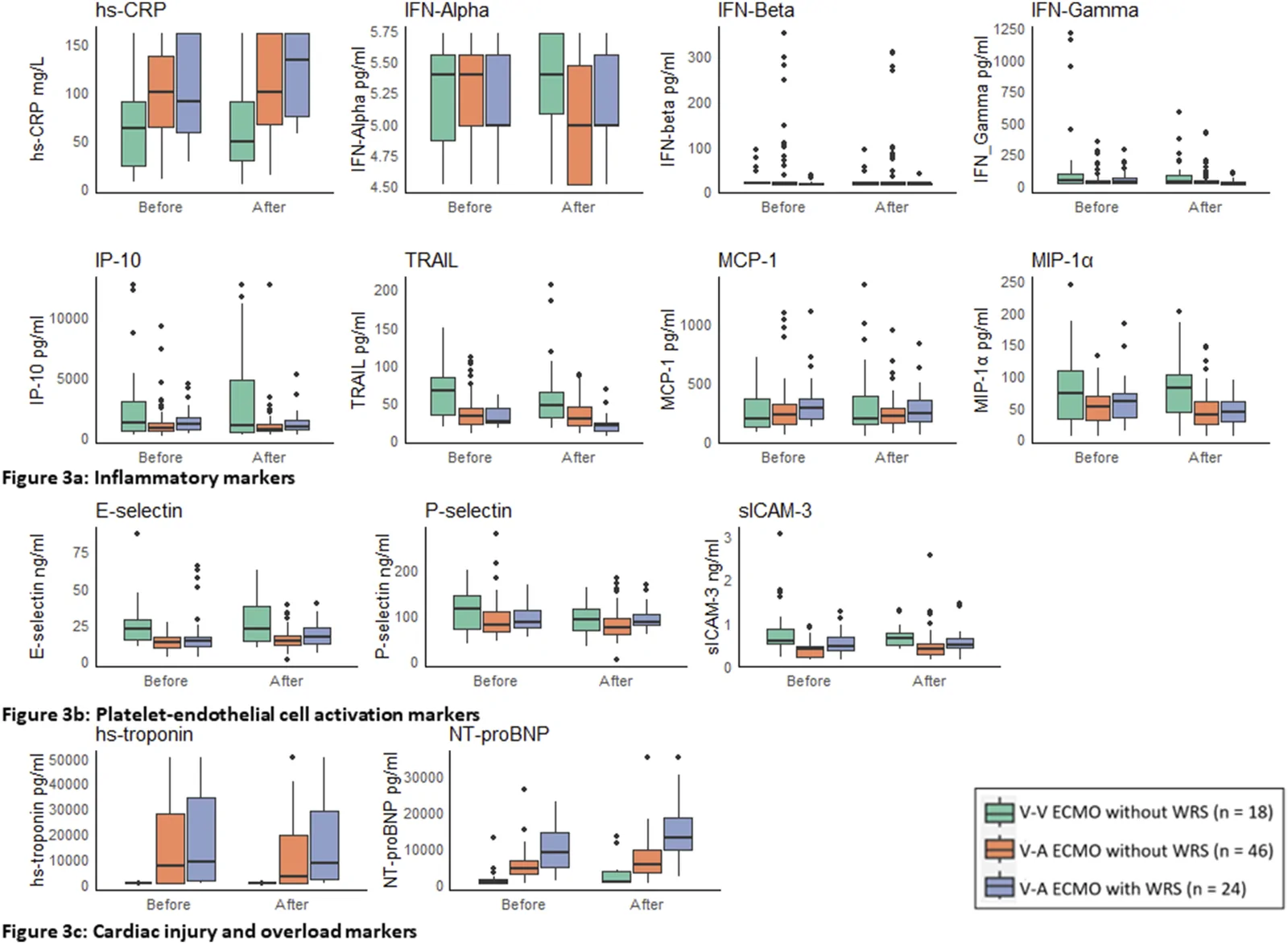
(a–c) Markers of inflammation, endothelial- and platelet activation and myocardial injury and overload stratified by WRS occurrence* Boxes represent medians and interquartile ranges (IQR). Whiskers indicate the range, with outliers plotted individually. E-Selectin = Endothelial Selectin Hs-CRP = High-sensitive C-Reactive Protein Hs-troponin = High-sensitive Cardiac Troponin I IFN-alpha = Interferon-alpha-2a IFN-beta = Interferon Beta IFN-gamma = Interferon Gamma IP-10 = Interferon-gamma-Inducible Protein 10 MCP-1 = Monocyte Chemoattractant Protein-1 MIP-1 = Macrophage Inflammatory Protein-10 NT-proBNP = N-terminal Pro-brain Natriuretic Peptide P-Selectin = Platelet Selectin sICAM-3 = Soluble Intercellular Adhesion Molecule-3 TRAIL = Tumor Necrosis Factor-Related Apoptosis-Inducing Ligand. *There were missing biobank samples on 25 out of 338 days (7%). Because no significant trends in MSD biomarker values within grouped periods (pre and post decannulation) were observed, all pre-decannulation and all post-decannulation biomarker values were aggregated. 255 × 190mm (96 × 96 DPI).

## Discussion

A significant proportion of patients who are being weaned from ECMO develop unanticipated WRS after decannulation, with older individuals and those supported with V-A ECMO following cardiac surgery or acute myocardial infarction being particularly at risk. Although subsequent fever and CRP responses could suggest that many of these WRS cases may be related to new-onset inflammation, this notion is not supported by the observed temporal dynamics of inflammatory and platelet-endothelial cell adhesion markers in our study. Instead, most individuals exhibiting WRS displayed markedly elevated NT-proBNP values before decannulation, with further increases thereafter. This finding suggests cardiac overload as a main determinant of WRS.

In our study, 75% of weaned patients exhibited clinical signs of new-onset inflammation, such as increasing CRP, leukocyte counts, or fever, following decannulation. Interestingly, while this was most common in WRS patients (92%), similar inflammatory responses were observed in both V-A and V-V ECMO patients without WRS (78% and 44%, respectively). These findings are consistent with existing literature, which report that up to 60% of weaned patients display symptoms such as fever and leukocytosis.^7,17,18^ Notably, despite the increase in clinical signs of inflammation in weaned patients, there was no correlation with rising levels of inflammatory biomarkers after decannulation in our study.

This observation can be explained in several ways. First, CRP is a nonspecific marker involved in the acute phase response to a multitude of stimuli and clinical conditions.^19,20^ The lack of significant changes in key inflammatory cytokines typically associated with infection, such as the interferons involved in immune responses against intracellular pathogens, suggests that factors other than infection are driving the observed increase in CRP. Second, the increased incidence of fever after decannulation does not necessarily indicate inflammation, as ECMO actively maintains body temperature around 36°C. It has been postulated that a shift in hypothalamic setpoint after a period of active temperature management during ECMO support may explain the elevated temperatures observed post-decannulation.^9^ However, others argue that active cooling does not directly affect the thermoregulatory center but rather triggers defensive mechanisms that generate and conserve heat.^21^ It is possible that these compensatory responses persist after decannulation, resulting in fever without an inflammatory or infectious cause. This phenomenon bears similarities to fever observed after targeted temperature management for cardiac arrest, known as rebound hyperthermia.^22,23^ Although several theories have been proposed to explain rebound hyperthermia, the underlying pathophysiological mechanisms remain unclear.^22,24,25^

The most striking observation in our study was the highly significant increase in NT-proBNP concentrations in patients with WRS. This finding suggests that increased myocardial loading conditions after ECMO decannulation are an important contributor to its development. Notably, this rise was most pronounced in patients who clinically appeared to have septic, rather than cardiogenic, shock. Patients with limited myocardial reserve may be particularly susceptible to the adverse effects of inflammation on cardiac function once circulatory ECMO support is discontinued.^20^ Indeed, circulating proinflammatory cytokines have been shown to exert negative inotropic effects on cardiac myocytes by altering intracellular Ca^2+^ homeostasis.^26,27^ Moreover, increasing temperatures and/or the development of fever post-decannulation can exacerbate heart failure by increasing heart rates and metabolic demands.^28^

In contrast to NT-proBNP, there was no significant surge in troponin levels in V-A ECMO patients with WRS compared to V-A ECMO patients without. This suggests that myocardial loading, but not injury plays a key role in WRS. The release of troponin could be the result of increased inflammation and coagulation, as studies indicate that troponin release is common in patients with activation of these pathways.^29,30^

The intricate interactions between inflammatory processes, increased cardiac wall stress, and cardiomyocyte dysfunction complicates the determination of their precise contributions to WRS development.^20^ However, in the absence of clear differences in measured biomarkers of inflammation and platelet-endothelial cell activation between patients with and without WRS, the observed change in NT-proBNP concentrations suggests that it is not inflammation, but cardiac overload what distinguishes patients with WRS from those without. Moreover, previous studies did not find new-onset inflammation after decannulation to be associated with increased mortality.^7,9,17^ This substantiates the notion that the high mortality rates observed in WRS patients are primarily due to cardiac failure.

Patients who developed WRS exhibited significantly higher NT-proBNP values even before decannulation, as well as a (non-significant) trend towards higher fluid balances. This could indicate suboptimal timing of ECMO decannulation in some. Indeed, others have previously suggested that NT-proBNP could be a valuable prognostic for evaluating V-A ECMO patients prior to weaning.^31^ Moreover, the increased use of inotropic support prior to decannulation among WRS patients may reflect a more limited myocardial reserve which, together with the vasodilatory effects of these drugs, warrants particular attention during weaning decisions. Currently, there are no standardized guidelines for assessing cardiac function after ECMO decannulation.^32^ Consequently, cardiac biomarkers and echocardiographic parameters are not systematically monitored, even though CRP, leukocyte counts, and temperature are evaluated daily. This may lead physicians to disregard potential cardiac dysfunction when clinical signs of inflammation arise. Therefore, we stress the need for prompt evaluation of cardiac function and volume status in patients who develop WRS even if clinical signs suggest an inflammatory cause for hemodynamic instability.

Our study has several limitations. The sample size was low, specifically in the V-V ECMO group, and the patients heterogeneous, which may limit generalizability of our findings and underscores the need for larger, multicenter studies to confirm the results of this exploratory study. Furthermore, patients were enrolled over a 12-years time-period during which ECMO care evolved considerably, including a growing use of percutaneous closure devices for decannulation and the adoption of stricter weaning protocols from 2018 onwards. Following these changes, we observed a significantly reduced cumulative fluid balance and a non-significant trend reduction in WRS incidence after decannulation. Since our findings suggests that cardiac overload contributes significantly to WRS development, future weaningmight be further improved by incorporating comprehensive hemodynamic monitoring data (e.g., pulmonary artery catheter measurements, central venous pressure and echocardiographic data). Lastly,, sample degradation over time may have affected biomarker stability, potentially influencing the findings.^33^ However, limited knowledge exists on this issue,^34,35^ and duplicate measurements for each timepoint partly mitigated these effects.

## Conclusions

WRS is a frequent complication after liberation from ECMO and associated with high mortality. Its occurrence exclusively after V-A support, alongside marked NT-proBNP surges post-decannulation, points to heart failure from excessive wall stress and volume overload as a key factor in the etiology of WRS – despite frequent signs of fever and new or worsening systemic inflammation suggesting otherwise. Larger studies with comprehensive assessments of cardiac contractility and loading conditions shortly before and after V-A ECMO decannulation are needed to confirm this hypothesis.

## Supplemental material

Supplemental material - Exploring weaning-related shock: Cardiac injury and overload, platelet-endothelial cell activation, and inflammatory response markers following ECMO decannulation Supplemental material for Exploring weaning-related shock: Cardiac injury and overload, platelet-endothelial cell activation, and inflammatory response markers following ECMO decannulation by Lara C.A. Pladet, Carlos V. Elzo Kraemer, Stefan Nierkens, Jeannine A. Hermens, Emma Rademaker, Lennie P.G. Derde, Christiaan L. Meuwese, Dirk W. Donker, Olaf L. Cremer in Perfusion

## Data Availability

Data is available upon request.*
