# Supplementary material for: Exploring weaning-related shock: Cardiac injury and overload, platelet-endothelial cell activation, and inflammatory response markers following ECMO decannulation
Source: Perfusion. 2026 Apr 6;41(8):1453–62. doi: 10.1177/02676591261439583 (PMC13736300; doi:10.1177/02676591261439583)
Supplement: Supplementary file 1 [file sj-pdf-1-prf-10.1177_02676591261439583.pdf]

Supplementary material

Table of Contents Supplementary material 1

Supplementary File 1: ECMO weaning protocols 2

Supplementary Figure 1a: 4

Supplementary Figure 1b 4

Supplementary Table 1: incidence of WRS in postcardiotomy patients according to surgery type ... 5

Supplementary Figure 2 6

Supplementary Figure 3a-c. 7

Supplementary Table 2 9

References 10

Supplementary File 1: ECMO weaning protocols

Weaning from V-A ECMO occurred via a structured weaning algorithm comprising multiple steps (1).

Before considering weaning, the following clinical conditions must be met:

Clinical Conditions for Initiating Weaning:

- The etiology of the heart failure includes an expectation of (partial) recovery of myocardial function.
- There is a hemodynamically stable situation for at least 24 hours, defined as: pulse pressure (pulsatility) >10 mmHg, mean arterial pressure >65 mmHg, lactate <1.5 mmol/L, and SVO_2 >65%. This stability is achieved without, or with only low doses of, inotropes/vasopressors.
- The patient is in a euvolemic state.
- There is radiological resolution of pulmonary edema under relatively low ventilatory settings (FiO_2 <40% on the ventilator, PEEP <8–10 cm H_2O).
- There is no liver failure.

If these conditions are met, a weaning attempt is conducted in a multidisciplinary setting. The team includes an intensivist, cardiac surgeon, heart failure cardiologist, perfusionist, and, if necessary, a cardiac anesthesiologist.

Preferably, hemodynamic monitoring during V-A ECMO is performed using a Swan-Ganz catheter.

Echocardiographic parameters for successful weaning:

- A left ventricular ejection fraction (LVEF) > 20-25% with minimal ECMO support (≤1.5 L/min)
- A velocity time integral of the left ventricular outflow tract (VTI LVOT) > 10 with minimal ECMO support (≤1.5 L/min)
- A lateral mitral annulus peak systolic velocity >6 cm/sec with minimal ECMO support (≤1.5 L/min).

If a patient meets the above echocardiographic criteria, decannulation can be scheduled.

For V-V ECMO patients, the ventilation settings were adjusted to reach plateau pressures <30 cm H_2O and a FiO_2 <0.4. When this was reached, the sweep gas flow was reduced to 0 L/min for at least 24h, during which arterial blood gasses are monitored. If blood gasses were adequate and the patient remained clinically stable, removal of ECMO circuit was ensued.

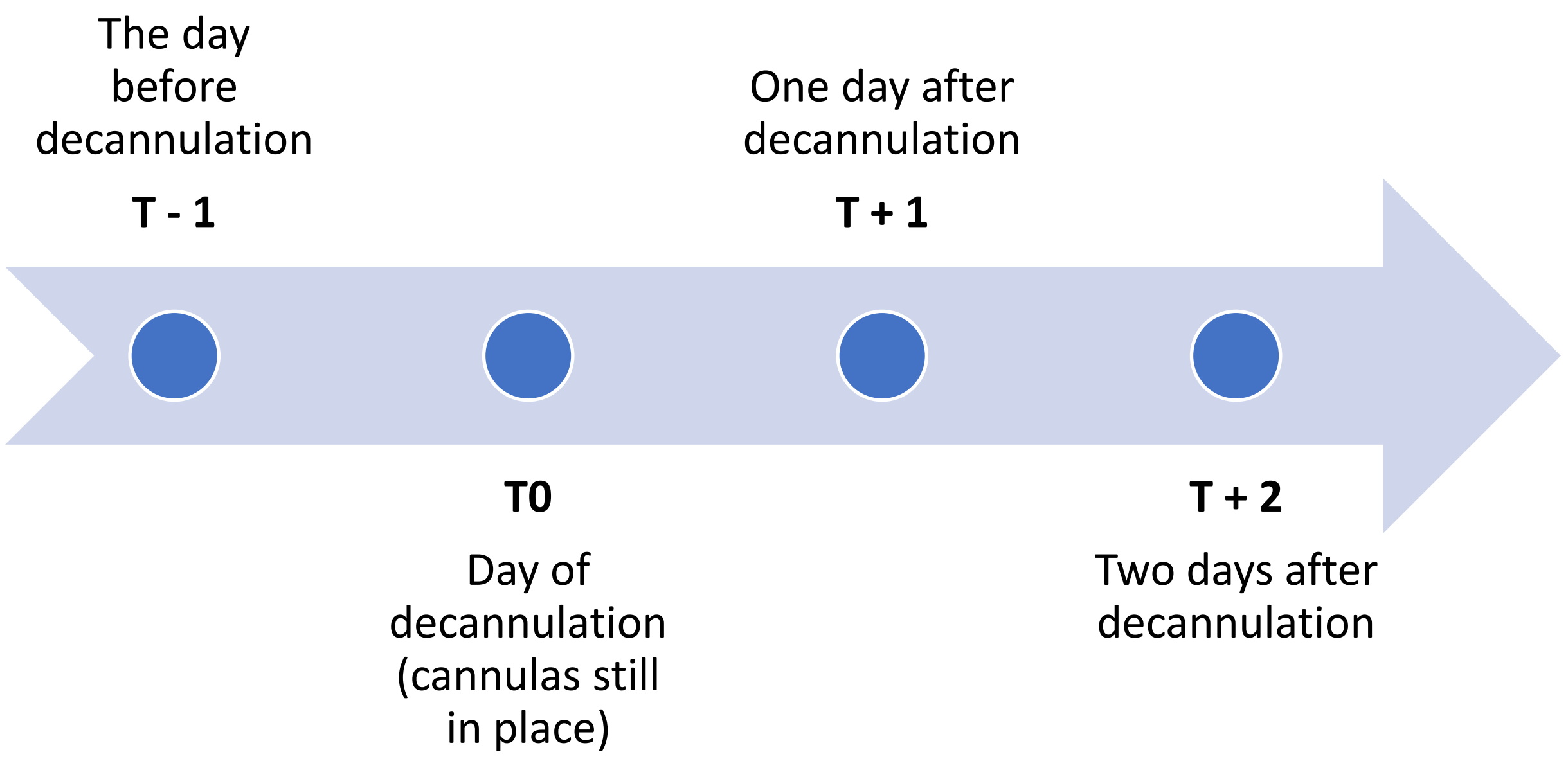

Supplementary Figure 1a: timing of biomarker measurements determined using the MSD platform (INF-α, INF-β, INF- γ, IP-10, TRAIL, MCP1, MIP-1 α, E-selectin, P-selectin, ICAM-3).

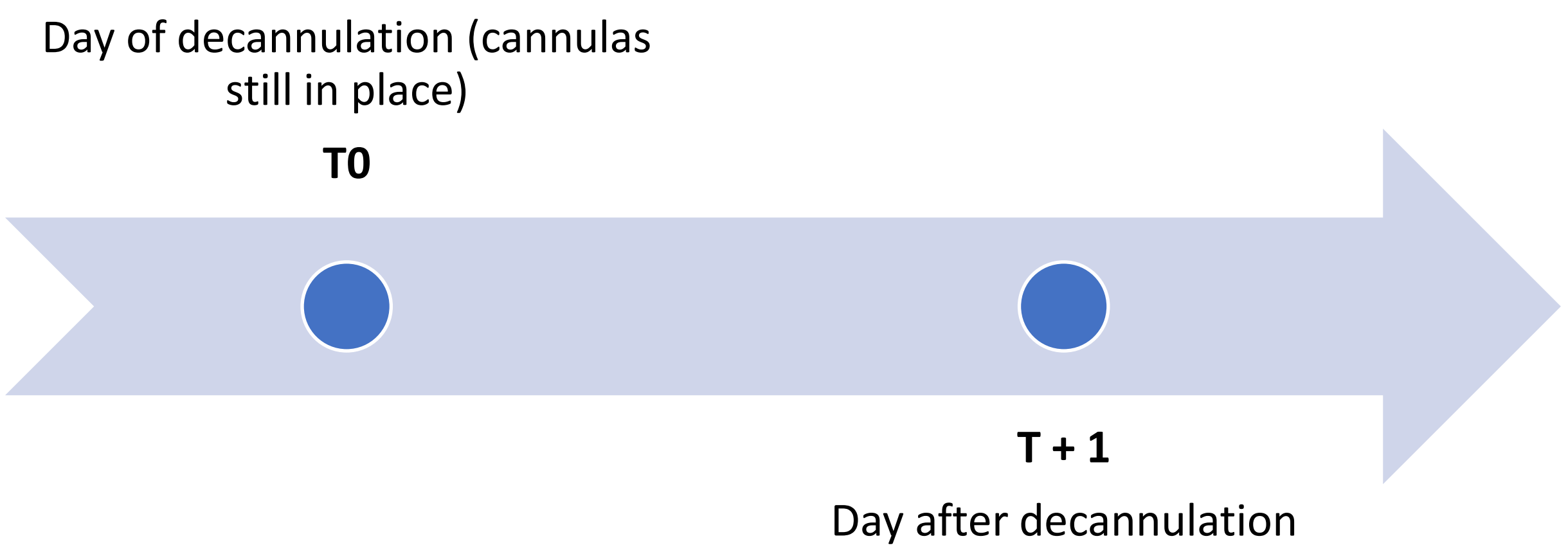

Supplementary Figure 1b: timing of biomarker measurements determined using regular diagnostic platforms (hs-CRP, HsTntI and NT-proBNP).

Supplementary Table 1: incidence of WRS in postcardiotomy patients according to surgery type

Surgery type	No WRS	WRS
**CABG only**	5	3
**CABG + MVP/MVR**	1	4
**CABG + AVR/AVP**	3	3
**MVP/MVR only**	2	1
**AVP/AVR only**	2	3
**Other***	4	2

CABG = Coronary Artery Bypass Grafting
MVP = Mitral valve plasty
MVR = mitral valve replacement
AVP = aortic valve plasty
AVR = aortic valve replacement
*Bentall, ascending aorta replacement

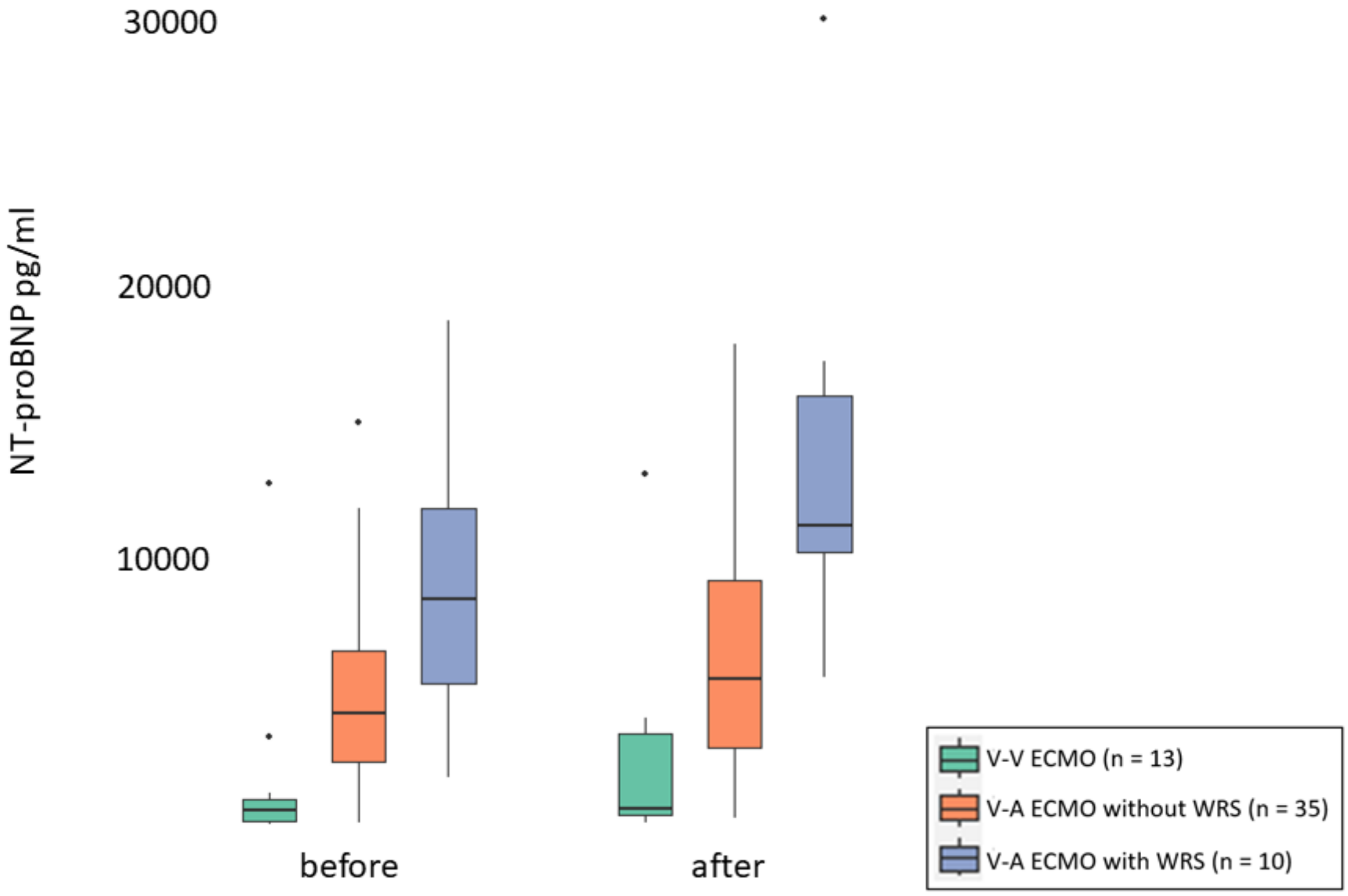

Supplementary Figure 2**: NT-proBNP values before and after decannulation in patients without AKI KDIGO 3**

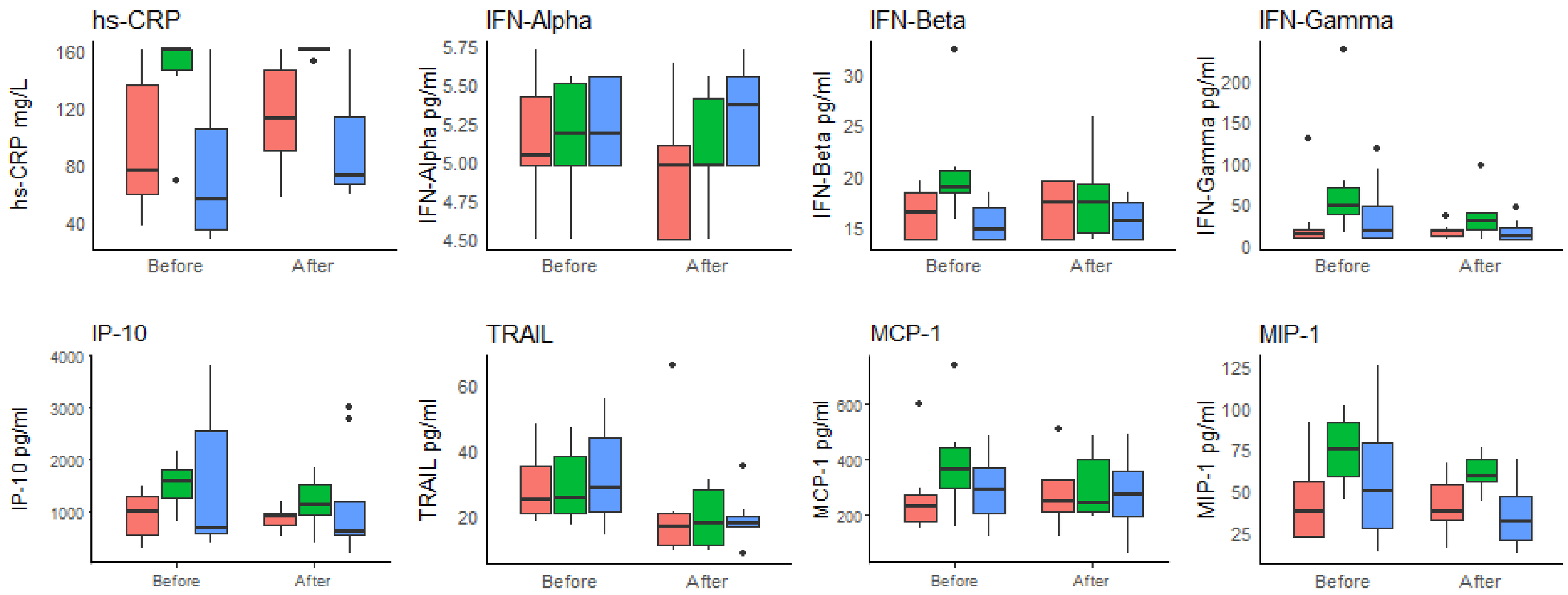

Figure 3a: inflammation markers according to presumed reason for WRS

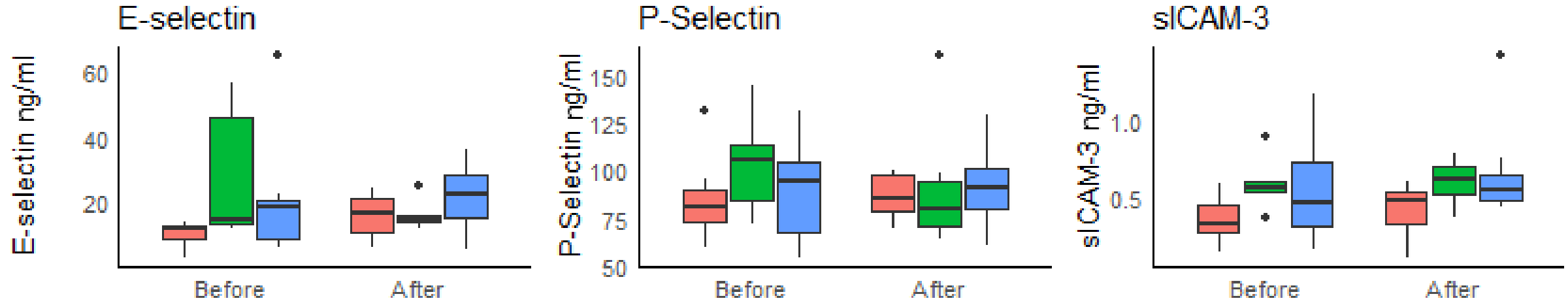

Figure 3b: endothelial- and platelet activation makers according to presumed reason for WRS

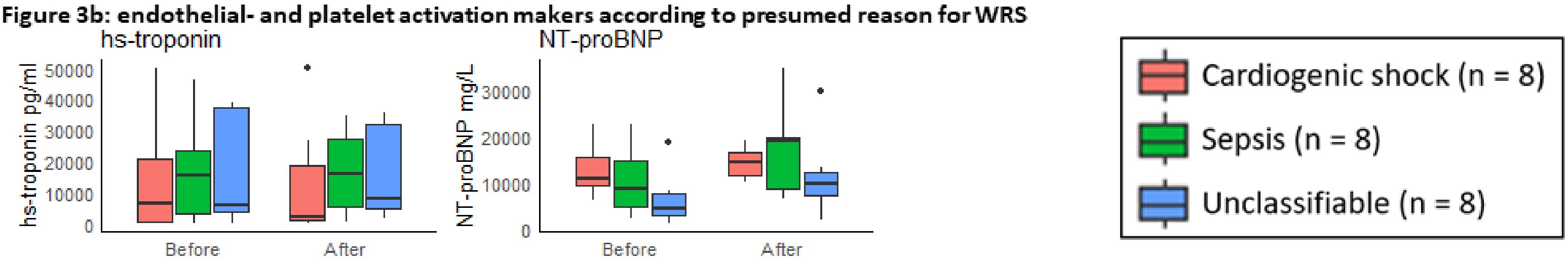

Figure 3c: cardiac injury and overload markers according to presumed reason for WRS

Supplementary Figure 3a-c. **Markers of inflammation, endothelial- and platelet activation and myocardial injury and overload stratified by presumed WRS etiology (according to clinical adjudication)**

Boxes represent medians and interquartile ranges (IQR). Whiskers indicate the range, with outliers plotted individually

E-Selectin = Endothelial Selectin
Hs-CRP = High-sensitive C-Reactive Protein
Hs-troponin = High-sensitive Cardiac Troponin I
IFN-alpha = Interferon-alpha-2a
IFN-beta = Interferon Beta
IFN-gamma = Interferon Gamma
IP-10 = Interferon-gamma-Inducible Protein 10
MCP-1 = Monocyte Chemoattractant Protein-1
MIP-1 = Macrophage Inflammatory Protein-10
NT-proBNP = N-terminal Pro-brain Natriuretic Peptide

P-Selectin = Platelet Selectin
sICAM-3 = Soluble Intercellular Adhesion Molecule-3
TRAIL = Tumor Necrosis Factor-Related Apoptosis-Inducing Ligand

Supplementary Table 2 incidence of WRS before and after 2018

Year	WRS patients (n)	Total patients (n)	WRS incidence	P-value
**2011 - 2017**	16	48	33%	
**2018 - 2022**	8	40	20%	0.099

References

1. Hermens JA, Meuwese CL, Szymanski MK, Gianoli M, van Dijk D, Donker DW. Patient-centered weaning from venoarterial extracorporeal membrane oxygenation: "A practice-oriented narrative review of literature". *Perfusion* 2023; 38: 1349-1359.
